# Greater brain activity during the resting state and the control of activation during the performance of tasks

**DOI:** 10.1038/s41598-019-41606-2

**Published:** 2019-03-22

**Authors:** Jie Huang

**Affiliations:** Department of Radiology, Michigan State University, East Lansing, MI 48824 USA

## Abstract

The brain’s operations are mainly intrinsic, involving the acquisition and maintenance of information for interpreting, responding to and predicting environmental demands. The brain’s on-going intrinsic activity (i.e., the resting-state activity) is spontaneous, but this spontaneous activity exhibits a surprising level of spatial and temporal organization across the whole brain. In this study we compared the intrinsic activity with the activity evoked by tasks, and made the comparison at several levels of analysis from a finger-tapping-activated area within the primary sensorimotor cortex to the whole brain. We found that, contrary to our intuition, the intrinsic activity was substantially larger than the task activity and consistently so for all levels of analysis. For the task state, the brain: (1) controlled the intrinsic activity not only during the performance of a task but also during the rest between tasks; (2) activated a task-specific network only when the task was performed but kept it relatively “silent” for other different tasks; and (3) simultaneously controlled the activation of all task-specific networks during the performance of each task.

## Introduction

Marcus Raichle explains the operations of the brain being mainly intrinsic, involving the acquisition and maintenance of information for interpreting, responding to and predicting environmental demands^1^. Traditional studies of brain function have mainly focused on task-evoked responses, but it is argued that the intrinsic activity may reflect the essence of brain function that accords well with the allocation of the brain’s energy resources^1^. The intrinsic activity accounts for 20% of all the energy consumed by the body, though the brain represents about 2% of total body weight. In contrast, task-evoked activities consume a surprisingly little additional energy, often less than 5%, suggesting that the brain’s enormous energy consumption is little affected by task performance^1^.

The brain’s on-going intrinsic activity (i.e., the resting-state activity) is spontaneous, but this spontaneous activity exhibits a surprising level of spatial and temporal organization across the whole brain^2,3^. The spatial organization of intrinsic activity appears to transcend levels of consciousness, being present under anesthesia in humans, monkeys, and rats and also during the early stages of sleep in humans^3–8^. These observations make it unlikely that the patterns of coherence and the intrinsic activity they represent are solely the result of unconstrained, conscious cognition (i.e., mind wandering or day dreaming)^3^. The observed spatial coherence of the intrinsic activity across the brain demonstrates the existence of functional connectivity as expressed in the maps of resting state coherence, and the intrinsic functional connectivity analyses also demonstrate the existence of large-scale brain functional connectivity networks^9,10^. Many functional network properties of the human brain have been identified during rest and task states, but the relationship between these two states have rarely been investigated^11^. In this study we compared the intrinsic activity with the activity evoked by tasks, and made the comparison at several levels of analysis from a finger-tapping (FT) activated area within the primary sensorimotor cortex to the whole brain.

We recently reported the discovery of functional areas of unitary pooled activity (FAUPAs) with fMRI^12^. A FAUPA is defined as an area in which the temporal variation of the activity is the same across the entire area, i.e., the pooled activity is a dynamically unitary activity. As an example, consider performing a FT task. Simultaneously tapping five fingers of the right-hand should induce a dynamic response in the well-confined five-finger representation area of the left primary motor area M1, and the temporal variation of this dynamic response should also be the same everywhere within this well-confined area, forming a FAUPA that is specifically associated with the FT task. We used new techniques to identify FAUPAs that involved the iterative aggregation of voxels dependent upon their intercorrelation^13^. The determination of FAUPA is objective and automatic with no requirement of a priori knowledge of the activity-induced ideal response signal time course, and this method enables us to identify FAUPAs that are associated with a specific task^12^. All task-associated FAUPAs form a network specific for the task, and these task-specific networks enable us to compare network activity between the task state and the resting state.

A recent fMRI study of an achiasmic human visual cortex quantifies the relationship between the fMRI BOLD signal and neural response; the magnitude of a stimulus-induced BOLD response is proportional to approximately 0.5 power of the stimulus-evoked underlying neural response^14^. We assessed intrinsic activity with a 12-min resting-state (rs) fMRI and activity evoked by tasks with a 12-min task-fMRI. During the rs-fMRI the participants were instructed to not think of anything but remain awake. During the task-fMRI they performed three tasks of word-reading (WR), pattern-viewing (PV) and FT. The neural activity was measured as the BOLD signal change squared at each time point, and was subsequently compared between the intrinsic activity and the task activity.

## Results

### Activity at the level of a FT-activated area within the primary sensorimotor cortex

**Figure 1:**
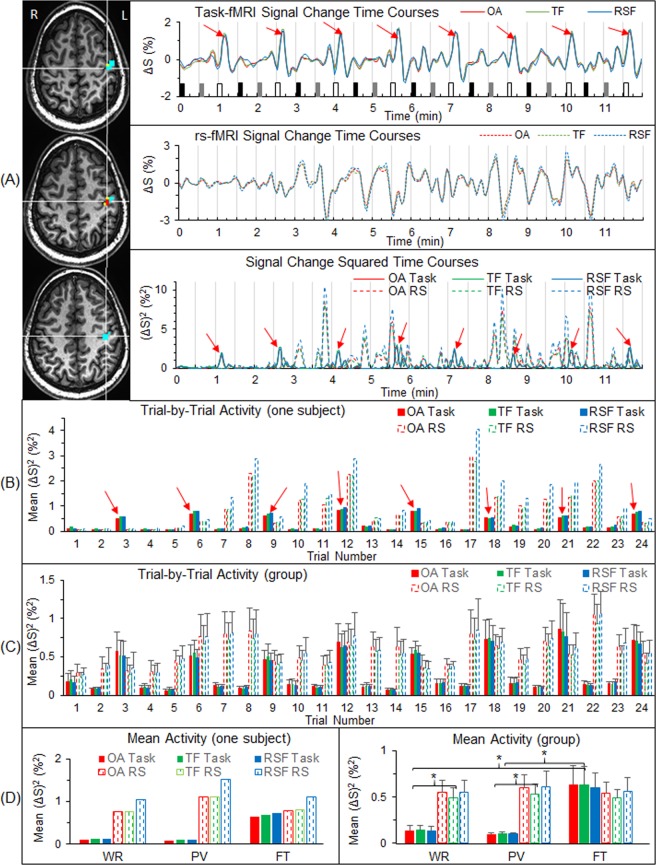
Comparison between the task activity and the intrinsic activity at the level of voxels. (**A**) For a representative participant, the three images show a task-fMRI FAUPA (TF) and a rs-fMRI FAUPA (RSF) with an overlapped area (OA) between them in the left primary sensorimotor cortex. The TF comprises the three yellow voxels plus the two red voxels of the OA, and the RSF comprises the fourteen cyan voxels plus the two red voxels. The three solid lines in the top panel show the time courses of the BOLD signal change of the task-fMRI for the three areas (green for TF, blue RSF and red OA) in the top plot. The task paradigm consisted of 24 trials shown by the 24 bars: black bars representing word-reading, gray bars pattern-viewing and white finger-tapping. Each task lasted 6 s followed by 24 s rest. The three dashed lines in the middle panel show the time courses of the BOLD signal change of the rs-fMRI for the three areas, and reflect the intrinsic activity in these areas during the 12-min resting-state. The bottom panel shows the time courses of the square of the BOLD signal change for both task-fMRI (solid lines) and rs-fMRI (dashed lines). Eight red arrows point out the large signal changes associated with the underlying neural activity evoked by the finger tapping task^14^. The three dashed lines represent their corresponding time courses of the intrinsic activity measured with the rs-fMRI. (**B**) For the same participant, the solid bars represent the task activity averaged over each trial period for the 24 trials, and the dashed bars represent the corresponding intrinsic activity for the corresponding periods. (**C**) As B averaged for the nine participants. (**D**) As B averaged over the eight trials of each task type for the representative participant (left) and the corresponding average for the group (right). For each of the three areas (TF, RSF and OA), the intrinsic activity was significantly larger than the task activity for both WR and PV (*: paired t-test, the maximal p < 0.01). The bar indicates the corresponding standard error of the mean across the participants.

We compared intrinsic activity with task-evoked activity within the primary sensorimotor cortex. Using the task-fMRI, for each participant we identified a FAUPA in the primary sensorimotor cortex (Fig. 1A), and examined the activity within that area from trial to trial (Fig. 1B,C). Compared to the WR and PV tasks, the activity within the FAUPA showed the expected activation on each of the eight FT trials, an activation significantly larger than that during WR or PV (paired t-test, maximal p < 0.01) (Fig. 1D, right). The intrinsic activity was measured during the 12-min rs-fMRI, and this 12-min time period was further divided into 24 time periods with each period of 30 s, creating 24 pseudo task trials that correspond to the 24 trials of the task-fMRI. For the representative participant, the intrinsic activity remained low during the first 3 minutes and then increased dramatically and irregularly for the last 9 minutes (Fig. 1A, middle and bottom plots). It demonstrated an irregular variation (Fig. 1B,C), but its magnitude was similar for all the three pseudo tasks and similar in magnitude to the FT-evoked activation (Fig. 1D, right). This intrinsic activity, however, was significantly larger than the task activity during both WR and PV (paired t-test, maximal p < 0.01), showing a significantly *lower* activity during the task performance of WR or PV relative to the corresponding intrinsic activity during rest. A further analysis with (1) a FAUPA identified from the rs-fMRI that partially overlapped the FAUPA identified from the task-fMRI and (2) the area of overlap between these two FAUPAs both produced similar results (Fig. 1).

### Activity at the level of seven selected task-associated FAUPAs

**Figure 2:**
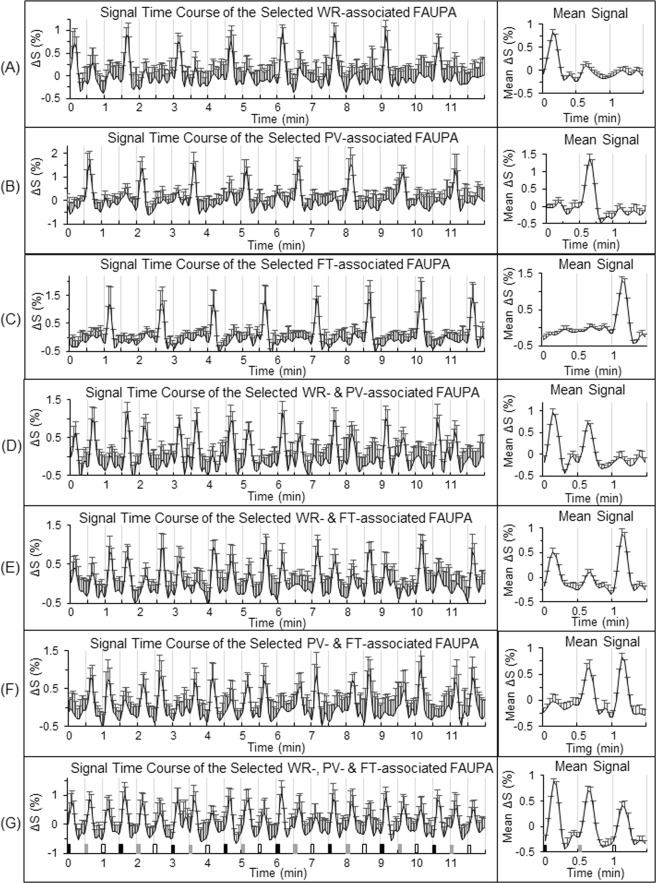
Illustration of the seven selected task-associated FAUPAs. A task-associated FAUPA is defined as a FAUPA that is activated when the task is performed^12^. There are three tasks of WR, PV and FT in the task-fMRI, and therefore there are total of seven functional groupings (i.e., seven categories) among these three tasks as illustrated from (**A**) to (**G**). For each participant, a representative task-associated FAUPA was selected for each category. The left plot in each panel represents the time course of the group-mean signal change of the selected FAUPA for that category. For each participant, the trial-mean signal change averaged over the eight trials was computed first for the three tasks, and then the group-mean of this trial-mean signal change was compared for the three tasks (the right plot). The black, gray and white bars in (**G**) represent the onset and duration of WR, PV and FT, respectively. The error bar indicates the corresponding standard deviation across the participants.

**Figure 3:**
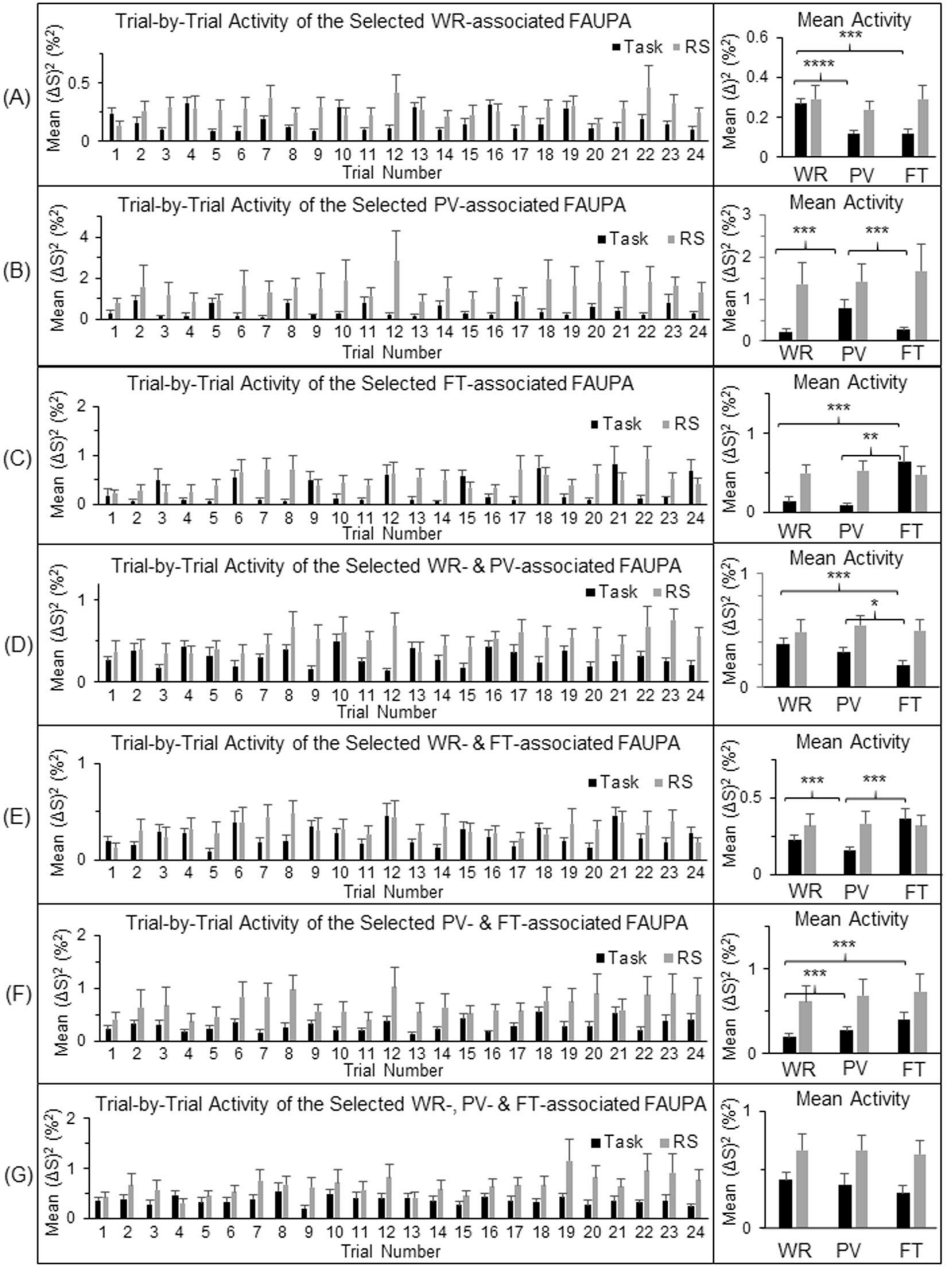
Comparison of the neural activity (BOLD signal change squared^14^) during the task-fMRI and the rs-fMRI at the level of the seven selected task-associated FAUPAs. For each of the seven functional groupings of the task-associated FAUPAs, the left plot shows the group-mean activity of the selected FAUPA from trial to trial for that category. For each participant and each category, the trial-mean activity averaged over the eight trials was computed first for the three tasks, and then the group-mean of this trial-mean activity was compared for the three tasks (the right plot). The intrinsic activity shown by the gray bars is consistently higher than the task-evoked activity throughout. The error bar indicates the corresponding standard error of the mean across the participants. *p < 0.05; **p < 0.01; ***p < 0.005; and ****p < 0.0001 (paired t-test).

There are three different tasks in this study, and therefore there are total of seven categories of the task-associated FAUPAs. To further compare the task-evoked activity with the intrinsic activity, for each participant we selected a representative task-associated FAUPA for each category and examined the activity from trial to trial (Figs 2 and 3). For each category except the one associated with all three tasks, the selected FAUPA was activated each time the task was performed but remained relative “quiet” for the other different task(s), as expected (Fig. 3, right). In contrast, the intrinsic activity during rest demonstrated an irregular variation from trial to trial for each selected FAUPA (Fig. 3, left), but its magnitude was similar for all the three pseudo tasks, regardless of the categories, as expected (Fig. 3, right). This magnitude, however, was *equal to or larger than* its corresponding task-evoked activation for each of the three tasks, again regardless of the categories, further demonstrating a substantially *suppressed* activity during the task performance for all the seven selected FAUPAs relative to the corresponding intrinsic activity during rest.

### Activity at the level of three task-specific networks

**Figure 4:**
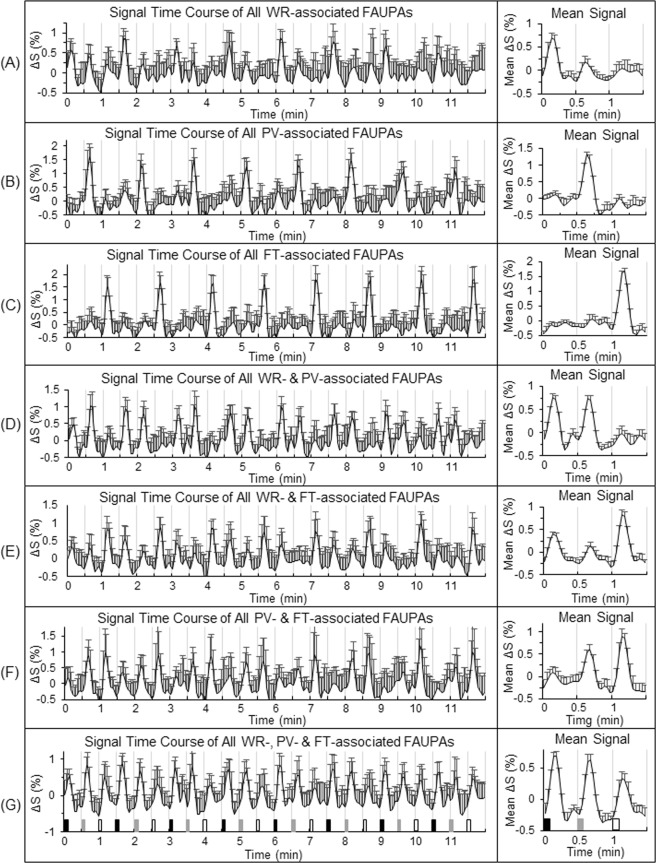
Illustration of the three task-specific networks. For each of the seven functional groupings illustrated in Fig. 2, for each participant the signal time course of the selected FAUPA was used to identify all task-associated FAUPAs for the category. The left plot in each panel represents the time course of the group-mean signal change of all identified task-associated FAUPAs for that category. For each participant, the trial-mean signal change was computed first for the three tasks, and then the group-mean of this trial-mean signal change was compared for the three tasks (the right plot). They are remarkably similar as those of the seven selected task-associated FAUPAs shown in Fig. 2. These task-associated FAUPAs were distributed across the whole brain, and could be further functionally grouped to compose task-specific networks. A task-specific network is defined as a set of FAUPAs in which each FAUPA is activated when the task is performed, and accordingly, there were three task-specific networks identified for the task-fMRI. The WR-specific network, for example, consisted of task-associated FAUPAs in the four functional groupings of (**A**,**D**,**E**,**G**), the PV-specific network was composed of FAUPAs in (**B**,**D**,**F**,**G**) and the FT-specific network comprised FAUPAs in (**C**,**E**,**F**,**G**), respectively. The WR-specific network was activated when each of the eight WR tasks was performed, but the other two networks remained relatively “quiet”, implying the separation of the three networks. The same was true of the other two task-specific networks. The error bar indicates the corresponding standard deviation across the participants.

**Figure 5:**
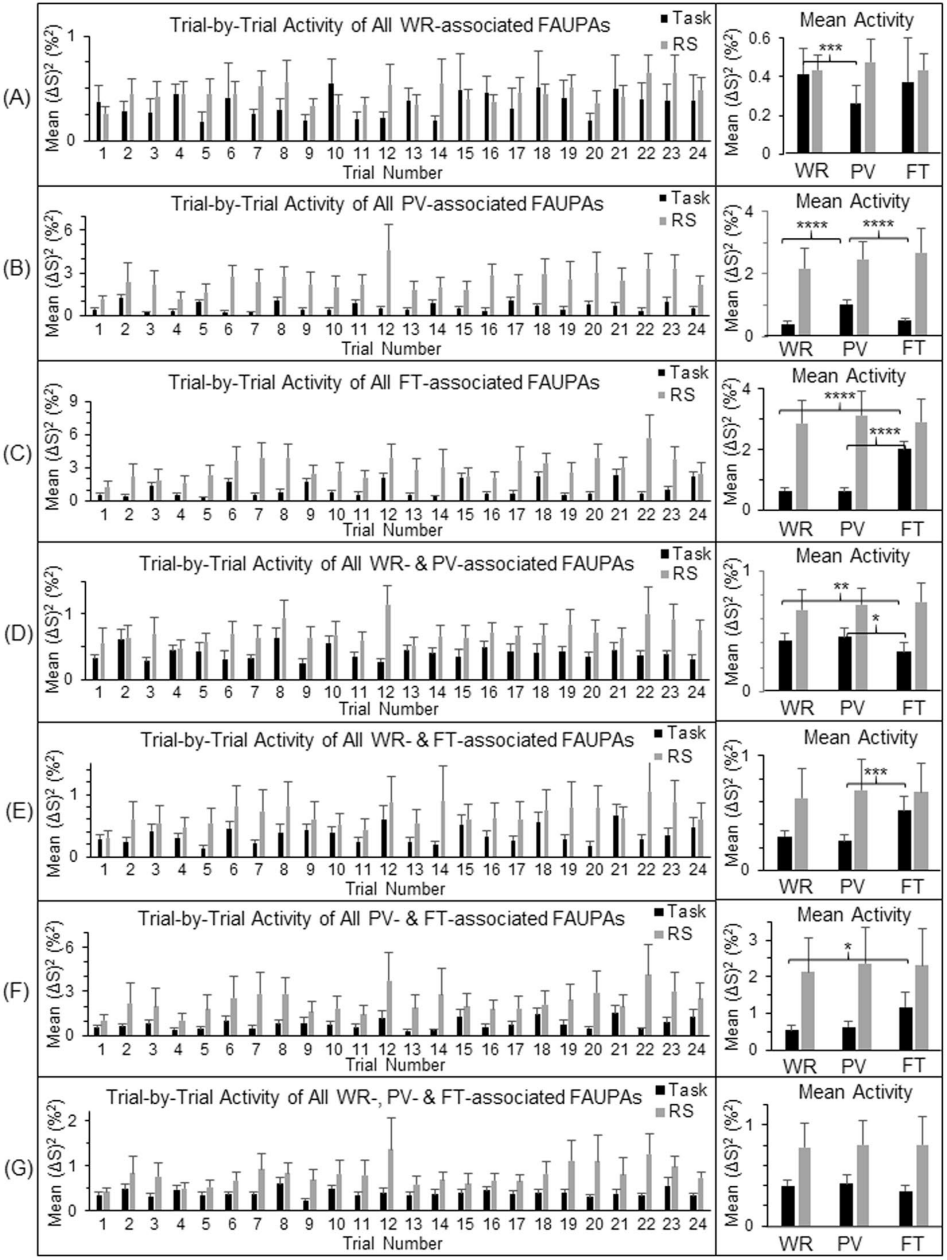
Comparison of the neural activity during the task-fMRI and the rs-fMRI at the level of the three task-specific networks. For each of the seven functional groupings of the task-associated FAUPAs, the left plot shows the group-mean activity of all identified task-associated FAUPAs from trial to trial for that category. For each participant and each functional grouping, the trial-mean activity was computed first for the three tasks, and then the group-mean of this trial-mean activity was compared for the three tasks (the right plot). For each of the three task-specific networks defined in the legend of Fig. 4, the activity from trial to trial is characterized by the corresponding activity of the four categories that compose the network. For example, the activity of the FT-specific network from trial to trial is characterized by the activity of the categories (**C**,**E**,**F**,**G**). This network was activated each time the FT task was performed but remained relatively “quiet” for both the WR and PV tasks. The intrinsic activity shown by the gray bars is consistently higher than the task-evoked activity throughout. The error bar indicates the corresponding standard error of the mean across the participants. *p < 0.05; **p < 0.01; ***p < 0.005; and ****p < 0.0001 (paired t-test).

To further compare the task-evoked activity with the intrinsic activity, we identified three task-specific networks defined in the legend of Fig. 4 for each individual participant, and examined the activity of each of the three networks from trial to trial (Fig. 5). The group-mean ± standard deviation of the total number of the task-associated FAUPAs were 68 ± 81, 78 ± 47 and 97 ± 49 for the WR-, PV- and FT-specific networks, respectively. These task-associated FAUPAs distributed across the whole brain, forming the functional networks specific for the tasks. For each task-specific network, that network was activated each time the task was performed but remained relatively “quiet” for the other two different tasks, as expected. For example, the activation of the FT-specific network was greater during the FT task than when the WR and PV tasks were performed (Fig. 5C,E,F). Both the WR- and PV-specific networks showed a similar behaviour of the task-evoked activity as that of the FT-specific network, demonstrating a dynamically controlled activation of the brain during the performance of these three different tasks. The intrinsic activity during rest, on the other hand, demonstrated an irregular variation from trial to trial for each network (Fig. 5, left), but its magnitude was similar for all the three pseudo tasks, regardless of the networks, as expected (Fig. 5, right). This magnitude, however, was *equal to or larger than* its corresponding task-evoked activation for each of the three tasks, again regardless of the networks, further demonstrating a substantially *suppressed* activity during the task performance for all the three networks relative to the corresponding intrinsic activity during rest.

### Activity at the whole brain level

**Figure 6:**
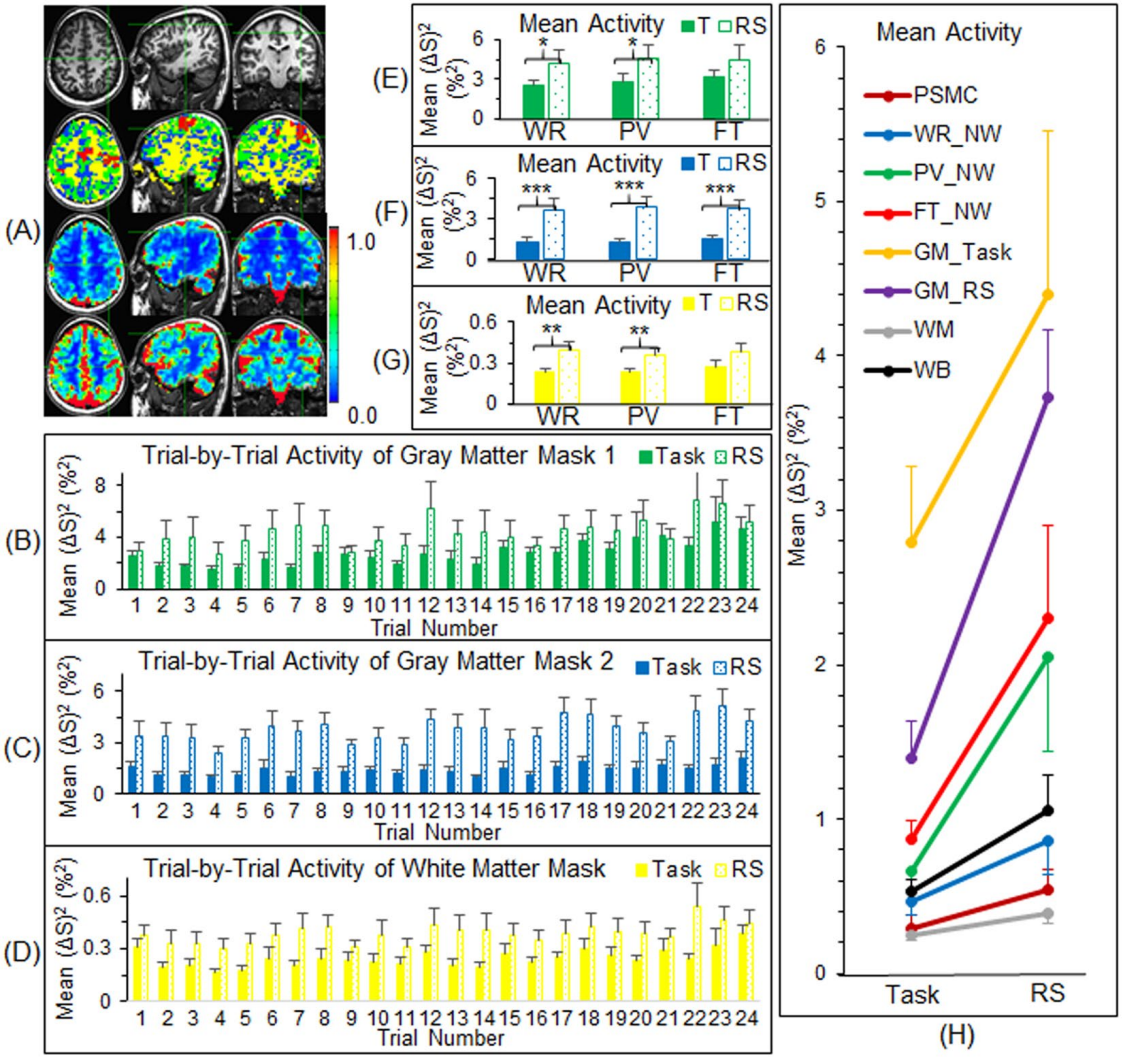
Comparison of the neural activity between the task-fMRI and the rs-fMRI at the level of gray matter, white matter and whole brain. (**A**) The three images in the second row illustrate four masks represented by four colours (only three slices in the representative participant are selected for illustration): (1) the red clusters represent the network mask that consisted of the three task-specific networks; (2) the green clusters represent the first mask of the gray matter that consisted of all FAUPAs identified with the task-fMRI, excluding the network mask; (3) the blue clusters represent the second mask of the gray matter that consisted of all FAUPAs identified with the rs-fMRI, excluding all FAUPAs identified with the task-fMRI; and (4) the yellow clusters represent the white matter mask that consisted of the whole brain mask, excluding all FAUPAs identified with both task- and rs-fMRI. The images in the third row and in the last row show the overall temporal mean of the task activity and of the intrinsic activity, respectively. The colour bar represents the magnitude of activity (%^2^). (**B**–**D**) The trial-by-trial activity for the first gray matter mask, the second gray matter mask and the white matter mask, respectively. Note that the intrinsic activity is consistently larger than the task activity throughout. (**E**–**G**) The activity averaged over the eight trials for the first gray matter mask, the second gray matter mask and the white matter mask for the three tasks, respectively. (T: task; *p < 0.05; **p < 0.02; ***p < 0.0005; paired t-test.) (**H**) Comparison of the overall mean activity (averaged for all time points) between the task activity and the intrinsic activity from the level of voxels to the whole brain. The intrinsic activity is about twice that of the task activity at each level, and the mean and standard deviation of their relative differences were 116 ± 57 (%). PSMC: primary sensorimotor cortex; NW: network; GM: gray matter; WM: white matter; and WB: whole brain. The error bar indicates the corresponding standard error of the mean across the participants.

We also compared the magnitude of activity between the task-fMRI and the rs-fMRI at the other areas that were *not* associated with any of the three tasks. As expected, both the task activity and the intrinsic activity demonstrated an irregular variation from trial to trial, but the magnitude of the intrinsic activity was equal to or larger than that of the task activity for each trial for both gray matter and white matter (Fig. 6B–D). [The FAUPAs identified for each participant for both task- and rs-fMRI are mainly located within the gray matter, and the total number of the FAUPAs for the rs-fMRI is significantly larger than that for the task-fMRI^12^. Accordingly, two gray matter masks were generated for the comparison (Fig. 6B,C).] The task activity of the gray matter, excluding those FAUPAs that were functionally grouped into the three task-specific networks as a result of the task performance, showed no difference among the three tasks (Fig. 6E,F). Its magnitude, however, was substantially lower than that of the intrinsic activity during rest, further demonstrating a substantially suppressed activity of the gray matter during the task performance. For the white matter, both the task and intrinsic activity showed the similar behaviours but at approximately ten times lower magnitude than that of the gray matter (Fig. 6G). For both the gray and white matter areas that were not associated with any of the three tasks, the neural activity of the task state was significantly smaller than that of the resting state (Fig. 6E–G). Finally, the magnitude of the temporal mean intrinsic activity was about twice that of the temporal mean task activity at each level from the FT-activated area at the primary sensorimotor cortex to the whole brain (Fig. 6H), demonstrating a substantially suppressed activity of the whole brain during the task performance relative to the intrinsic activity during rest.

## Discussion

The brain’s intrinsic activity, measured with the 12-min rs-fMRI in which the participants were instructed to not think of anything but remain awake during the whole scan, was compared with the task activity, measured with the 12-min task-fMRI in which the participants performed three tasks in sequence: word reading, pattern viewing and finger tapping. These tasks were performed repeatedly eight times with an interval of 24 s between each task. As expected, each of the eight FT tasks induced a large BOLD signal change at the primary sensorimotor cortex (Fig. 1A), and this FT-evoked activation was significantly larger than the activity measured during the periods of either WR or PV (paired t-test, maximal p < 0.01) (Fig. 1D, right). The intrinsic activity at the same area, however, was at the same level as that of the FT-evoked activation, and significantly larger than the activity measured during the periods of either WR or PV (paired t-test, maximal p < 0.01) (Fig. 1D, right). Evidently, this intrinsic activity was significantly suppressed during the whole 12-min task-fMRI both during task performance and during the rest in between, similarly for all three tasks, suggesting that the area was controlled to remain “silent” and activate only when the FT tasks were performed. The task-evoked activity showed a similar behaviour for each of the three task-specific networks, i.e., when a task was performed, the corresponding task-specific network was activated but the other two different networks were kept relatively “silent” (Fig. 5). This demonstrated a dynamically controlled activation of the three networks during the performance of each task. The corresponding intrinsic activity, as expected, showed no difference between the three tasks for each of the seven functional groupings of the task-associated areas (Fig. 5, right). Compared to the task-evoked activation, the mean intrinsic activity was equal to or substantially larger than the task-evoked activation, and again this intrinsic activity was substantially suppressed for each functional grouping during the whole 12-min task state. This showed a dynamically controlled operation of the brain for all three task-specific networks when performing these three different tasks. These results show that the brain: (1) controls the intrinsic activity not only during the performance of a task but also during the rest between tasks; (2) activates a task-specific network only when the task is performed but keep it relatively “silent” for the other two different tasks; and (3) simultaneously controls the activation of all the three task-specific networks during the performance of each task.

The intrinsic activity accounts for 20% of all the energy consumed by the body, and, in contrast, task-evoked activities consume less than 5% additional energy^1^, suggesting an additional task-evoked activity added on top of the intrinsic activity, consistent with our intuition. This study, however, found that the intrinsic activity was about twice the magnitude of the task activity at each level from the FT-activated area at the primary sensorimotor cortex to the whole brain (Fig. 6H). In addition to providing further evidence of a substantially suppressed intrinsic activity across the whole brain when performing the tasks, this substantially greater brain activity during the resting state supports the idea that the brain’s operations are mainly intrinsic, involving the acquisition and maintenance of information for interpreting, responding to and predicting environmental demands^1^. When performing tasks, the intrinsic activity across the whole brain is suppressed so the brain is more focused on the performance of the tasks and consumes less rather than more energy. A suppressed intrinsic activity during the task performance was observed for seven participants. The other two participants, however, showed a lower intrinsic activity during the resting state compared to the task activity during the task performance, reflected in a negative value of −30% for the mean difference between the intrinsic activity and the task activity for one participant (data not presented). The substantial variation in the intrinsic activity from participant to participant is also reflected in the substantially larger standard error of the mean of the intrinsic activity compared to that of the task activity at each level from the FT-activated area at the primary sensorimotor cortex to the whole brain (Fig. 6H). This result demonstrates an individually dependent intrinsic activity during the resting state. Intra-participant intrinsic activity could also vary substantially over time as evidenced in the representative participant (Fig. 1C). During the first three minutes of the resting state, the intrinsic activity was kept “silent” but increased substantially with time. These results suggest that the brain’s intrinsic activity is controlled by thought, but that control varies substantially over time and from participant to participant. For each individual people, being able to quantitatively assess the whole brain’s intrinsic activity during the resting state may offer a means of evaluating the intrinsic activity of the brain at different states such as during sleep, under anesthesia, in minimally conscious state, in coma, in vegetative state, or even in dead brain, potentially valuable in clinical diagnosis.

**Figure 7:**
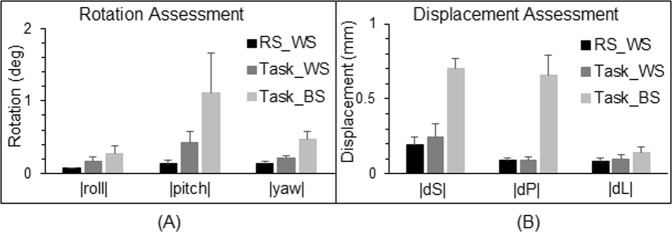
Comparison of the rotation and displacement between the rs- and task-fMRI scans. For the resting-state (RS) scan, the very first volume image was chosen as the base volume image (within-scan, WS), and for each of the six rigid-body co-registration parameter (three rotations and three displacements), the mean value of the absolute value of the parameter over the whole volumes was computed. Then, its corresponding group-mean value was computed and illustrated. For the task scan, we performed two assessments: (1) chosen the very first volume image of the task scan as the base (WS), and (2) chosen the very first volume image of the RS scan as the base (between-scans, BS). (**A**) Rotations around the three axes of the inferior-superior (roll), right-left (pitch), and anterior-posterior (yaw). |x|, the absolute value of x; deg: degree. (**B**) Displacements in the superior direction (dS), posterior direction (dP), and left direction (dL). The error bar indicates the corresponding standard error of the means.

Head motion could produce large signal changes, and to assess this motion effect we have compared the head motion between the rest and task scans. Figure 7 compares the 3-dimentional rotation and displacement between the rest and task scans. For each of the six rigid-body co-registration parameters, the group-mean value of the parameter was the smallest one for the rest scan compared to that of the task scan, showing that the larger signal variance during the rest scan could not arise from the motion artifact.

The brain anatomical structure varies substantially from subject to subject, but these variations do not affect the results of our group analysis. In the conventional fMRI analysis, each individual’s data are first converted to a standard template such as the Talairach space, and then the group analysis is conducted in this standard template. Accordingly, mismatched anatomical structures do affect the group analysis to certain degree. In this study, however, all our analyses were conducted in the original space. First, all FAUPAs were determined in the original space and then all task-associated FAUPAs were identified for each subject. For example, we first identified a FT-associated FAUPA in the primary motor area and then used its signal time course to identify all FT-associated FAUPAs for that subject. With the identified FT-associated FAUPAs, we computed their mean task-evoked activity and intrinsic activity from trial to trial for each individual subject. Finally, we conducted the group analysis of these mean task-evoked activity and intrinsic activity from trial to trial for each category of the seven task-associated FAUPAs. Thus, the individual anatomical differences do not affect the results of this study. However, it remains to be explored how to conduct a FAUPA-based analysis in a standard template, considering the relative small size of FAUPAs.

One main purpose of this presented study is to compare the intrinsic activity with the task-evoked activity at the level of brain functional networks. The task paradigm consists of three different tasks of word reading, pattern viewing and finger tapping. These three tasks are associated with three different brain functional networks. The presented FAUPA method identified task-associated FAUPAs for each task type as well as their combinations (Fig. 4), enabling us to compare the intrinsic activity with the task-evoked activity for each of the three task-specific networks. It also enabled us to examine how the brain controlled the activation at the network level when performing these different tasks. It remains to be tested the extent of this control during the performance of tasks in general.

## Methods

This is a follow-up study of our previous study that reported the discovery of FAUPAs with fMRI^12^. Accordingly, the present study used the same subjects, same task paradigm, same image acquisition, similar image preprocessing procedures, and same algorithms for FAUPA determination. We have revised these paragraphs to make them concise. For more information please refer to our previous study^12^.

### Subjects

Nine healthy subjects (5 male and 4 female, ages from 21 to 55 years old with μ ± σ = 29.4 ± 11.1) participated in the study. The Institutional Review Board at Michigan State University approved the study, and written informed consent was obtained from all participants prior to the study. All methods were performed in accordance with the institution’s relevant guidelines and regulations.

### Task paradigm

The task paradigm consisted of a total of 24 task trials with 3 different tasks. Each trial was composed of a 6-s task period followed by a 24-s rest period. Task 1 was WR, task 2 was PV, and task 3 was FT. Participants were instructed to silently read each presented English word during a WR task period, passively view each presented pattern during a PV task period, and tap their right-hand five fingers as quick as possible at a random order during a FT task period. The visual stimulus was a black-and-white striped pattern, and the visual stimulation paradigm consisted of a total of 10 visual stimulation cycles (a 120 ms visual stimulus followed by a 480 ms blank screen for each cycle) during the 6-s task period^15^. During the 24-s rest period, participants were instructed to focus their eyes on a small fixation mark at the center of the blank screen and try not to think of anything.

### Image acquisition

Functional brain images were acquired on a GE 3.0 T clinical scanner with an 8-channel head coil using a gradient echo Echo-Planar-Imaging pulse sequence (TE/TR = 28/2500 ms, flip angle 80°, FOV 224 mm, matrix 64 × 64, slice thickness 3.5 mm, and spacing 0.0 mm). Thirty eight axial slices to cover the whole brain were scanned, and the first three volume images were discarded. For each participant, the center frequency, the transmit gain and the two receive gains were kept the same for the task- and rs-fMRI scans to ensure the same baseline for both scans. The visual stimuli were projected onto a vertical screen placed inside the magnet bore using a MR-compatible Hyperion digital projection system, the stimulation presentation was controlled by a PC equipped with E-Prime, and a BrainLogic Fiber Optic Button Response System with a pair of 5-button MR-compatible keypads was used to record participants’ finger-tappings (Psychology Software Tools, Inc., Pittsburgh, PA). The participants viewed the screen through a mirror mounted on top of the head coil. For the participants who needed vision correction, MR-compatible lenses were used. Head movement was minimized by restraint using tape and cushions. Each participant first had a 12-min resting-state (rs) fMRI scan and then a 12-min task fMRI scan. Each scan yielded a total of 288 volume images (total time points N = 288). For the rs-fMRI scan, the participants were instructed to close their eyes and try not to think of anything but remain awake during the whole scan. After the task-fMRI scan, T1-weighted whole-brain MR images were also acquired using a 3D IR-SPGR pulse sequence.

### Image preprocessing

Image preprocessing of the functional images was performed using AFNI (http://afni.nimh.nih.gov/afni)^16^, including: (1) removing spikes; (2) slice-timing correction; (3) motion correction; (4) spatial filtering with a Gaussian kernel with a full-width-half-maximum of 4.0 mm; (5) computing the mean volume image; (6) bandpassing the signal intensity time courses to the range of 0.009 Hz–0.08 Hz; and (7) computing the relative signal change ΔS (%) of the bandpassed signal intensity time courses. ΔS = [S(t) − S_0_] × 100/S_0_ (%), where S(t) is the signal time course and S_0_ is the mean of S(t). (The motion correction used the “3dvolreg” routine in AFNI, and the very first volume image of the resting-state scan was chosen as the base volume image for both resting-state and task scans). After these preprocessing steps, further image analysis was carried out using in-house developed Matlab-based software algorithms.

### FAUPA determination

A statistical model and Matlab-based software algorithms were developed and tested to determine FAUPA, and FAUPAs were identified and reported for both the rs-fMRI and task-fMRI^12,13^. The FAUPA determination involved the iterative aggregation of voxels dependent upon their intercorrelation, and the algorithms were described in detail in our previous study^12^. The determination consists of two major procedures. (1) Using a first statistical criterion the algorithm first identifies a stable region-of-interest (ROI) in which the signal time courses of all voxels show a similar temporal behaviour; and (2) Using a second statistical criterion it determines whether this stable ROI satisfies the condition of being a FAUPA by comparing the temporal behaviour of signal time course of the voxels within the FAUPA with those bordering the FAUPA.

### Identification of task-associated FAUPAs and task-specific networks

We define a task-associated FAUPA as a FAUPA that is activated when performing the task. To identify those FAUPAs that are associated with a task, we first generate a task-induced ideal response by convolving the temporal paradigm of the task with a hemodynamic response function, using the 3dDeconvolve program in AFNI with the convolution kernel SPMG3. We then compute its relative signal change time course by subtracting the signal time course with its mean and dividing that change with the mean. This ideal response time course is then used to identify those FAUPAs that are associated with the task. There are three different tasks in this study, and therefore there are total of seven combinations among these tasks: (1) FAUPAs associated with the WR task alone; (2) those associated with the PV task alone; (3) those with the FT task alone; (4) those with both WR and PV tasks; (5) those with both WR and FT tasks; (6) those with both PV and FT tasks; and (7) those with all the three tasks (Fig. 2). Accordingly, seven ideal response time courses are generated for these seven categories of task-associated FAUPAs. As task-induced responses may vary from participant to participant, for each participant, to identify a representative task-associated FAUPA for each category, we first compute the R of the ideal response time course with the signal time course of each FAUPA, and then sort them with R. Then, we visually examine the BOLD signal time courses for those FAUPAs with large R values, and select the FAUPA with a signal time course that most resembles the ideal response to be the representative task-associated FAUPA for that category and that participant. To identify task-associated FAUPAs for each category and each participant, we use the signal time course of the representative FAUPA to compute its R with that of all other FAUPAs, and identify those FAUPAs with R > 0.8 as the task-associated FAUPAs for the category and the participant. We define a task-specific network consisting of all FAUPAs that are activated when performing the task. Accordingly, there are three networks specifically for the three tasks of WR, PV and FT, respectively, as described in the legend of Fig. 4.

## Data Availability

Both the original and processed fMRI images plus final research data related to this publication will be available to share upon request with a legitimate reason such as to validate the reported findings or to conduct a new analysis.
